# Tuberculosis cohort analysis in Zimbabwe: The need to strengthen patient follow-up throughout the tuberculosis care cascade

**DOI:** 10.1371/journal.pone.0293867

**Published:** 2023-11-08

**Authors:** Tariro Christwish Mando, Charles Sandy, Addmore Chadambuka, Notion Tafara Gombe, Tsitsi Patience Juru, Gerald Shambira, Chukwuma David Umeokonkwo, Mufuta Tshimanga

**Affiliations:** 1 Department of Primary Health Care Sciences, Family Medicine, Global and Public Health Unit, University of Zimbabwe, Harare, Zimbabwe; 2 National TB and Leprosy Control Unit, Ministry of Health and Child Care, Harare, Zimbabwe; 3 African Field Epidemiology Network, Harare, Zimbabwe; 4 African Field Epidemiology Network, Monrovia, Liberia; The University of Georgia, UNITED STATES

## Abstract

**Introduction:**

Globally people with tuberculosis (TB) continue to be missed each year. They are either not diagnosed or not reported which indicates possible leakages in the TB care cascade. Zimbabwe is not spared with over 12000 missed cases in 2020. A preliminary review of TB treatment outcomes indicated patient leakages throughout the presumptive cascade and undesirable treatment outcomes in selected cities. Chegutu District had pre-diagnosis and pretreatment losses to follow-up while Mutare City among others had 22.0% of outcomes not evaluated in the second quarter of 2021, and death rates as high as 14% were recorded in Gweru District. The problem persists despite training on data analysis and use. The TB cohorts were analysed to determine the performance of the care cascade and the spatial distribution of treatment outcomes in Zimbabwe.

**Methods:**

Using data from district health information software version 2.3 (DHIS2.3), a secondary data analysis of 2020 drug-sensitive (DS) TB treatment cohorts was conducted. We calculated the percentage of pre-diagnosis, and pre-treatment loss to follow-up (LTFU). For TB treatment outcomes, ‘cured’ and ‘treatment completed’ were categorized as treatment success, while ‘death’, ‘loss to follow-up (LTFU), and ‘not evaluated’ were categorized as undesirable outcomes. Univariate analysis of the data was conducted where frequencies were calculated, and data was presented in graphs for the cascade, treatment success, and undesirable outcomes while tables were created for the description of study participants and data quality. QGIS was used to generate maps showing undesirable treatment outcomes.

**Results:**

An analysis of national data found 107583 people were presumed to have TB based on symptomatic screening and or x-ray and 21.4% were LTFU before the specimen was investigated. Of the 84534 that got tested, 10.0% did not receive their results. The treatment initiation rate was 99.1%. Analysis of treatment outcomes done at the provincial level showed that Matabeleland South Province had the lowest treatment success rate of 77.3% and high death rates were recorded in Matabeleland South (30.0%), Masvingo (27.3%), and Matabeleland North (26.1%) provinces. Overall, there were high percentages of not-evaluated treatment outcomes.

**Conclusion:**

Pre-diagnosis LTFU was high, and high death and loss to follow-up rates were prevalent in provinces with artisanal and small-scale mining (ASM) activities. Unevaluated treatment outcomes were also prevalent and data quality remains a challenge within the national TB control program. We recommended strengthening patient follow-up at all levels within the TB care cascade, strengthening capacity-building for data analysis and use, further analysis to determine factors associated with undesirable outcomes and a study on why LTFU remains high.

## Introduction

Globally 1.5 million people died from TB out of the 10 million people who were infected with TB in the year 2020 [1]. Zimbabwe remains one of the top 8 countries in Africa on the world’s top 30 list of countries heavily burdened by **TB/HIV and MDR-TB.** The high-burden countries account for 85–89.0% of TB globally [2]. In Zimbabwe, treatment outcomes have progressively improved to an 84.0% success rate by 2018 but still fall short of the 90.0% national target [3].

TB treatment cohort review process involves reviewing information on the patient’s clinical status, treatment outcome, adequacy of the medication regimen, treatment adherence or completion, directly observed therapy (DOT) status, and results of contact investigation [4]. Cohort analysis can also provide information on contributing factors to either successful or unfavorable treatment outcome [5]. In Zimbabwe, TB treatment cohort analysis is performed at the health facility level and the findings are entered into the quarterly reporting form. The cohort analyses are performed quarterly, and the cohort analyzed is those clients enrolled during the same period the previous year for drug-sensitive TB and previous two years for drug-resistant TB. The aggregate data is then submitted to the district level where they are entered into district health information software version 2.3 (DHIS2.3) [3]. The cohort review process in Zimbabwe categorizes treatment outcomes in two main groups which are treatment success (cured/completed), and undesirable outcomes (treatment failure, loss to follow-up, and not evaluated) [6].

According to the Global TB Report of 2021, an estimated 12891 cases were missed in Zimbabwe during the year 2020 [7]. Zimbabwe has a high proportion of losses to follow-up along the cascade and unevaluated TB treatment outcomes [8]. A preliminary review of cohort analyses in the District Health Information System 2.3 (DHIS2.3) indicated concerning percentages of leakages where 17% of presumed patients in Chegutu District did not have specimens sent to the laboratory for investigation and 11% of those who submitted specimens did not receive results. Furthermore, TB treatment outcomes were not evaluated as indicated in Mutare City (22.0%) in April-June 2021, against a target of (0.0%), approximately 40.0% of 2020 cohorts in Harare Uniformed Forces were not evaluated and 14% of patients lost their lives due to TB in Gweru District in the last quarter of 2020. Despite health worker trainings on making sense of TB data which encourage the importance of proper documentation and analysis of data, the percentage of not evaluated outcomes, loss of life and leakages within the TB cascade remains high. This affects the estimation of disease burden, planning for resource needs, and national target setting. The national TB program (NTP) is concerned about the treatment outcomes not being evaluated. This prompted an assessment of the TB treatment cascades and spatial distribution of treatment outcomes to inform programming and aid in crafting targeted interventions to the problems of leakages and unfavorable TB treatment outcomes).

## Materials and methods

### Study setting

Zimbabwe is a low to medium income southern African country with a population of 15 million [9]. The country has eight predominantly rural and two metropolitan administrative provinces. These provinces are further divided into 91 administrative districts from the recent delimitation activities [10]. However, there are still 73 health districts, which offer primary and secondary level care health services. There are four levels of care (primary, secondary, tertiary, and quaternary) within Zimbabwe’s health delivery system of which most services are offered at primary care level. The public sector (Ministry of Health and Child Care, local government, uniformed forces) provides the bulk (95.0%) of the health services and is complemented by private health service delivery system (profit and not-for-profit health institutions). Data analysed in this study were accessed from the public sector, and all patients diagnosed with TB from the private sector are referred to the public sector for notification and treatment. TB services (laboratory, and anti TB medicines) are offered for free in Zimbabwe, and are decentralized to peripheral health facilities [3].

Artisanal and small-scale miners are largely populated in Midlands province with a population of 19457 ASMS, followed by Mashonaland west 13267, Matabeleland South 12153, and Mashonaland central provinces [9]. Prevalence of TB in ASMs was 6.8% in Zimbabwe [11]. ASMs are highly mobile, work in remote areas with poor access to health services which contributes to late diagnosis which is associated with poor prognosis, and high mobility contributes to high losses to follow-up [12–14].

**Fig 1:**
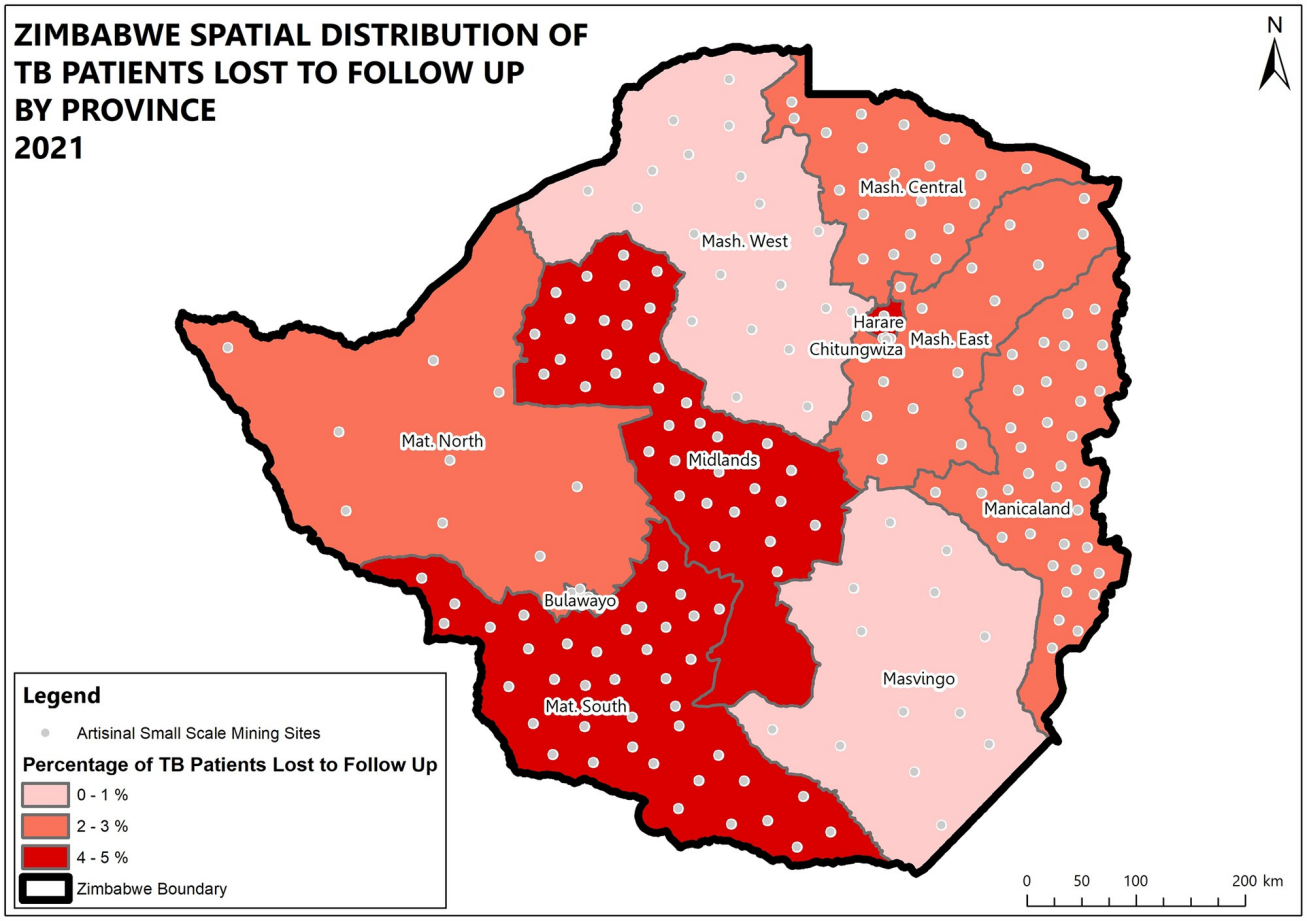
Schematic chart of TB surveillance in Zimbabwe.

Zimbabwe has 230 TB diagnosing sites across its 10 administrative provinces and a comprehensive TB surveillance system where data at all health facilities and community health workers are captured using paper-based tools (notification forms, community referral slips, and registers), consolidated into monthly and quarterly reporting forms. The data is forwarded to the district TB and leprosy coordinator (DTLC) for consolidation and forwarded to the district health information officer to enter the aggregate data into the electronic DHIS2.3. The data is reviewed and validated at district and provincial levels for completeness, accuracy, and consistency then cascaded to the national level and partners as indicated in Fig 1. The data is analyzed and used at every level of the surveillance system [15] (Fig 1). Reported aggregate data is based on predefined performance indicators. Some of the indicators are treatment success, cure, treatment completed, failure, death, lost to follow up, and not evaluated rates.

### Study design

We conducted a descriptive analysis of a secondary dataset of 2020 drug-sensitive TB treatment cohorts.

### Data source

The TB patient cohort aggregate data was obtained from DHIS2.3., an electronic database which records routine TB data. Treatment outcomes captured included treatment success, died, treatment failure, lost to follow up, not evaluated and cases notified DS-TB.

### Study population

The study population was 2020 DS-TB in Zimbabwe which was captured in DHIS 2.3. Treatment outcomes for DS-TB are evaluated after a year and was therefore reported in 2021.

### Study variables and data analysis

We analyzed variables for the presumptive cascade and these were, percentage of patients presumed to have TB based on symptomatic screening and or chest x-ray whose specimen was not sent for investigation, percentage of specimens with no results from the specimens sent for investigation, positivity rate which is percentage of positive results from those investigated, and treatment initiation which is percentage of cases initiated on treatment. At each stage of the cascade the percentage of patients lost to follow-up was calculated. We also analysed treatment outcomes and we calculated them as shown below.

Treatment outcomes:

$$\text{Treatment}\;\text{success}=\frac{Number\;of\;TB\;Cases\;with\;successful\;treatment\;outcome\;\left(cured\;and\;treatment\;completed\right)\times \;100}{Number\;of\;all\;forms\;of\;TB\;Cases\;with\;treatment\;outcomes\;analysed}$$

$$\text{Died}=\frac{\left(Number\;of\;deaths\;of\;all\;forms\;of\;TB\;during\;treatment\;\times 100\right)}{Number\;of\;all\;forms\;of\;TB\;Cases\;with\;treatment\;outcomes\;analysed}$$

$$\text{Treatment}\;\text{failure}=\frac{\left(Number\;\;of\;TB\;cases\;declared\;as\;treatment\;failure\;at\;the\;end\;of\;treatment\;\times 100\right)}{Number\;of\;all\;forms\;of\;TB\;Cases\;with\;treatment\;outcomes\;analysed}$$

$$\text{Lost}\;\text{to}\;\text{follow-up}=\frac{Number\;of\;all\;forms\;of\;TB\;cases\;who\;were\;lost\;to\;follow\;up\;during\;treatment\;\times \;100}{Number\;of\;all\;forms\;of\;TB\;Cases\;with\;treatment\;outcomes\;analysed}$$

$$\text{Not}\;\text{evaluated}=\frac{Number\;of\;TB\;cases\;whose\;outcome\;was\;not\;documented\;in\;the\;register\;at\;the\;end\;of\;treatment\;\times \;100}{Number\;of\;all\;forms\;of\;TB\;Cases\;with\;treatment\;outcomes\;analysed}$$

The number of all forms of TB treatment outcomes analyzed was used as the denominator instead of the standard ‘number of all forms of TB cases registered’ when calculating percentages of DS-TB outcomes. This was done to cater for discrepancies noted between TB cases registered and TB cases with outcomes analyzed.

#### Operational definitions

Pre-diagnosis LTFU was defined as non-availability of test result (either smear microscopy or Xpert MTB/RIF) by the time person was reported as presumptive pulmonary TB patient. Pre-treatment LTFU was defined as non-registration for treatment after diagnosis by the time of reporting.

Data was exported from DHIS2.3 and data quality assessment was done first by profiling the data followed by cleaning where the inconsistencies were followed up and verified with respective provinces. Data was matched with manual quarterly reporting forms. Key informant telephone and face-to-face interviews were conducted to clarify and verify discrepancies in the numbers notified and the outcomes evaluated. Twelve key informants constituting of national and provincial monitoring and evaluation officers, health information officers, TB focal persons and TB and leprosy coordinators (TLC) were consulted. Question asked was “may you please shed light on the discrepancies between number notified and outcomes evaluated?”. All elected data was exported to Excel where it was cleaned. The data was presented as 11 provinces instead of the usual ten administrative provinces as we followed the NTP reporting format. Microsoft Excel was used conduct univariate analysis to calculate percentages of variables (pretreatment cascade and outcomes) and national treatment success rates. It was also used to generate graphs for the presumptive cascades, treatment success and undesirable outcomes for DS-TB. We used QGIS software to construct maps depicting undesirable treatment outcomes (death, lost to follow up, and not evaluated). A thematic analysis for the qualitative responses was conducted. The inductive approach to thematic analysis was used. The raw data was coded and then classified into three themes. These themes were then defined as data quality issues, outcomes not evaluated and incorrect reporting.

## Results

### Data quality

More males than females fell sick from DS-TB (64.1% males). Children constituted smaller percentages in DS-TB (5.6%) while the rest were adults. Of all who fell ill with TB 53.2% were bacteriologically confirmed, 39.1% were clinically diagnosed and the rest had extrapulmonary TB (Table 1).

**Table 1:** Demographic characteristics of notified DS-TB patients, 2020 cohorts, Zimbabwe.

	Pulmonary bacteriologically confirmed		Pulmonary clinically diagnosed		Extra pulmonary TB		
Age groups	Male	Female	Male	Female	Male	Female	Total (%)
<15	132 (2.3%)	126 (4.4%)	290 (7.5%)	272 (11.3%)	47 (6.5%)	34 (6.6%)	901 (5.6%)
15–64	5291 (92.6%)	2570 (90.5%)	3207 (84.4%)	1923 (79.9%)	602 (82.9%)	427 (82.9%)	14020 (87.2%)
65 +	292 (5.1%)	145 (5.1%)	374 (9.7%)	213 (8.8%)	77 (10.6%)	54 (10.5%)	1155 (7.2%)
**Total**	**5715 (100%)**	**2841 (100%)**	**3871 (100%)**	**2408 (100%)**	**726 (100%)**	**515 (100%)**	**16076 (100.0%)**

More (16151) cases had outcomes evaluated against 16076 notifications for drug-sensitive TB. The discrepancies were more pronounced at provincial level (Table 2).

**Table 2:** Discrepancies between DS-TB 2020 cases notified and outcomes evaluated, Zimbabwe.

Province	Total outcomes evaluated	Total cases notified	Discrepancy
Bulawayo	1439	1418	+21
Chitungwiza	516	557	-41
Harare	1656	1392	+264
Manicaland	1614	1820	-206
Mashonaland Central	1451	1469	-18
Mashonaland East	1216	1246	-30
Midlands	2290	2282	+8
Matabeleland North	954	999	-45
Matabeleland South	1145	1115	+30
Masvingo	1686	1603	+83
Mashonaland West	2184	2175	+9
**Total**	**16151**	**16076**	**+75**

From the key informant interviews, it was noted that the data discrepancies can be explained by data quality issues

*In our province there are three districts who reported one thing in DHIS2 yet their registers and quarterly report forms were saying something totally different*

(Key informant 8)

Health facilities were not evaluating treatment outcomes for patients they had transferred out who in turn were not evaluated by the receiving facilities.

Key informants 2 and 12

“*Facilities mainly in referral facilities forget to follow-up on treatment outcomes for patients they notified and immediately transferred out”*

In other provinces outcomes were evaluated, and the findings were either entered in wrong/ outdated datasets or they were not reported.

Key informant 1, 7 and 9

“*Recently recruited or trainee health information personnel are not sure which dataset to enter the information and end up entering into outdated one while some of them enter incorrect figures*”

*During one of the data driven support and supervisory visits we noted that the treatment outcomes were clearly documented in the registers but a few were reported on the quarterly report form which is then used to enter the data into DHIS2*”

### National TB presumptive cascade

**Fig 2:**
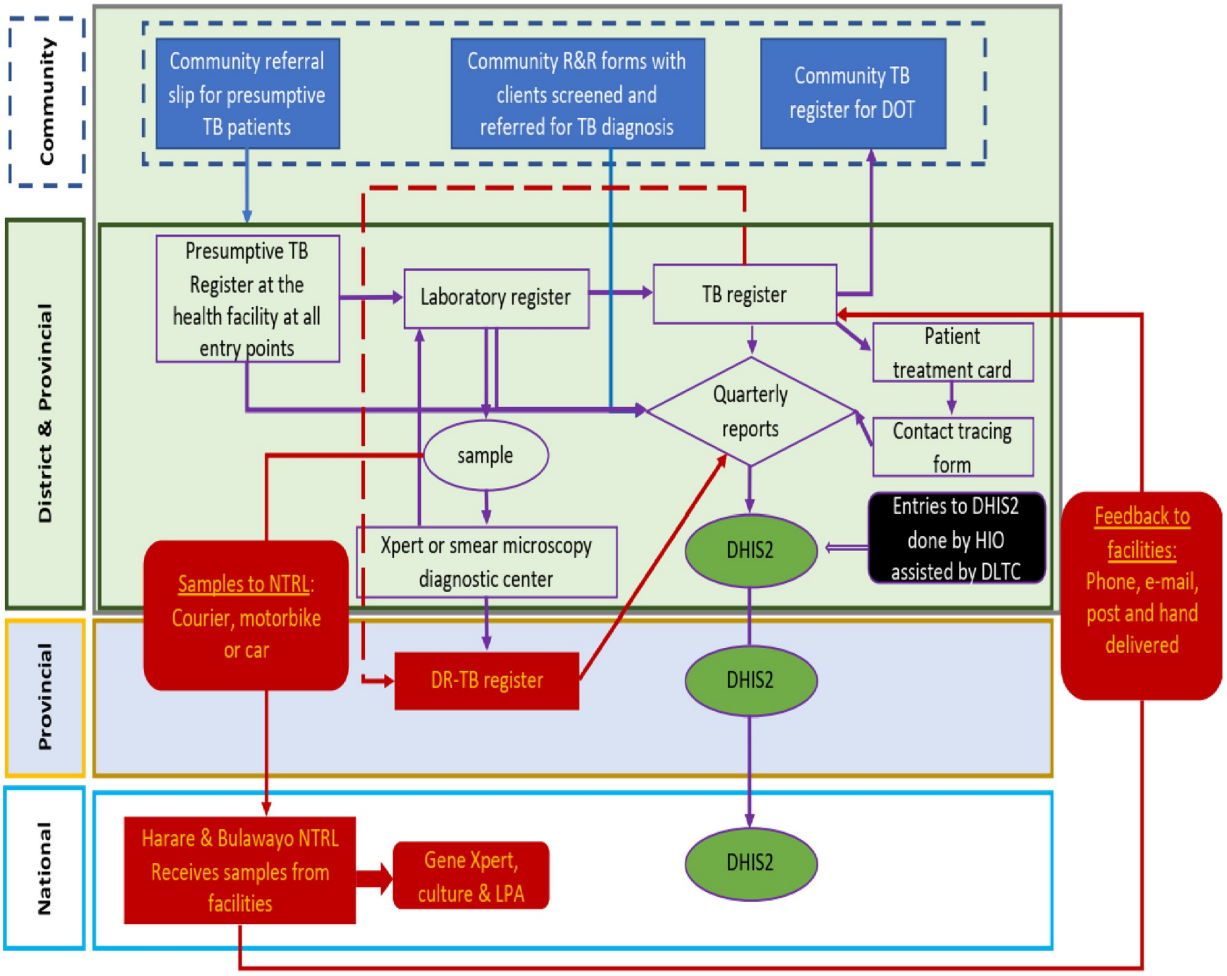
National presumptive TB cascade for the 2020 DS-TB Cohort, Zimbabwe.

There was a 21.4% loss to follow up between presumption of TB and specimen sent to the laboratory. Further to that, there was a 10.0% loss to follow up between specimen sent for investigation and results received which translates to overall pre-diagnosis loss to follow up of 29.3%. The positivity rate was 13.0%. Of the 9888 people who tested positive for TB, the program was able to initiate 9795 and this equates to a 99.1% treatment initiation rate. The target treatment initiation rate was 100%. (Fig 2).

**Fig 3:**
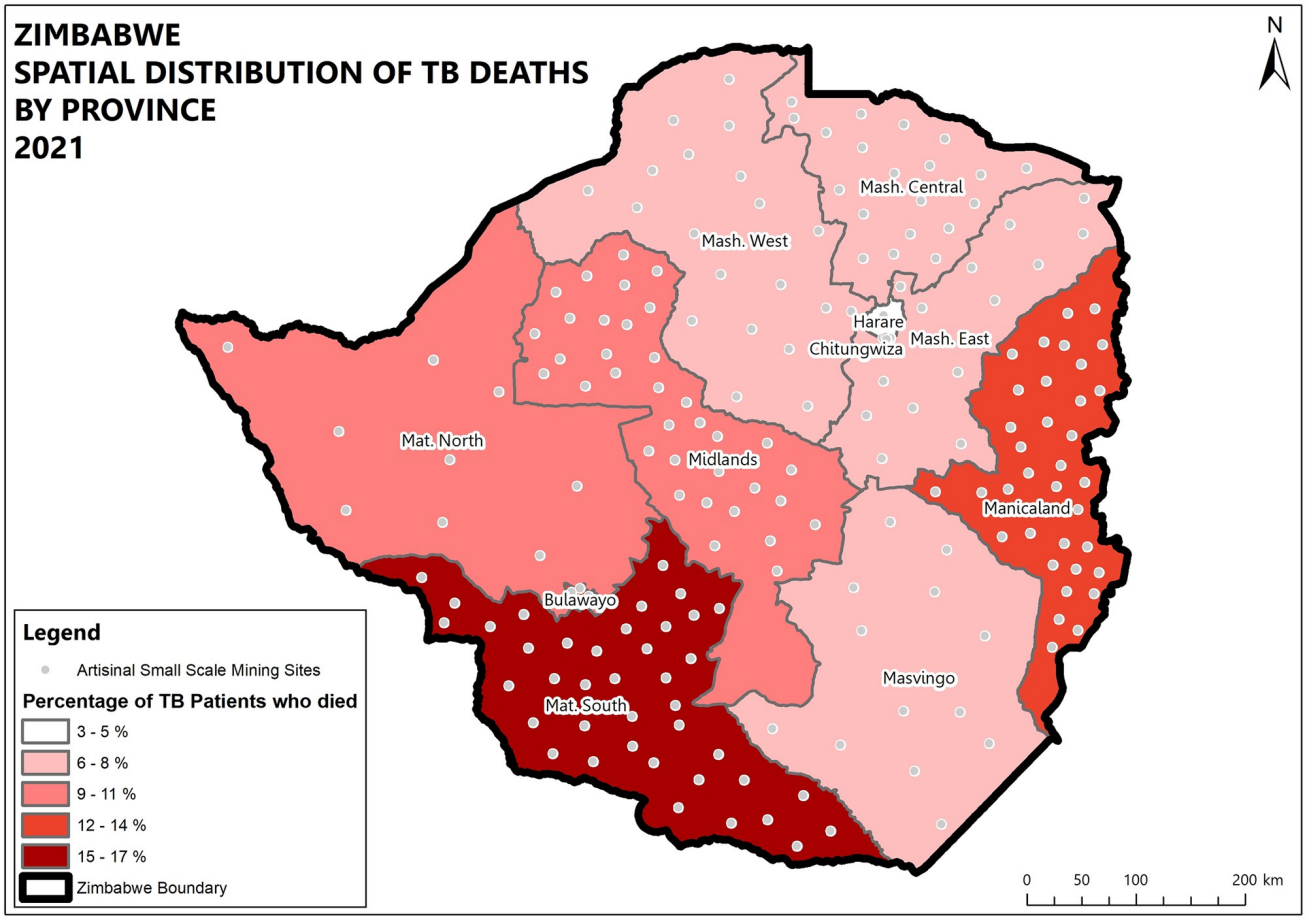
DS-TB treatment success outcomes for the 2020 cohort in Zimbabwe.

Provinces with the most successful treatment outcomes for DS-TB were Mashonaland West (94.7%), Masvingo (91.8%), and Harare (89.7%). The average national treatment success rate was at 87.7% which is above the 85.0% national target for 2021 (Fig 3).

**Fig 4:**
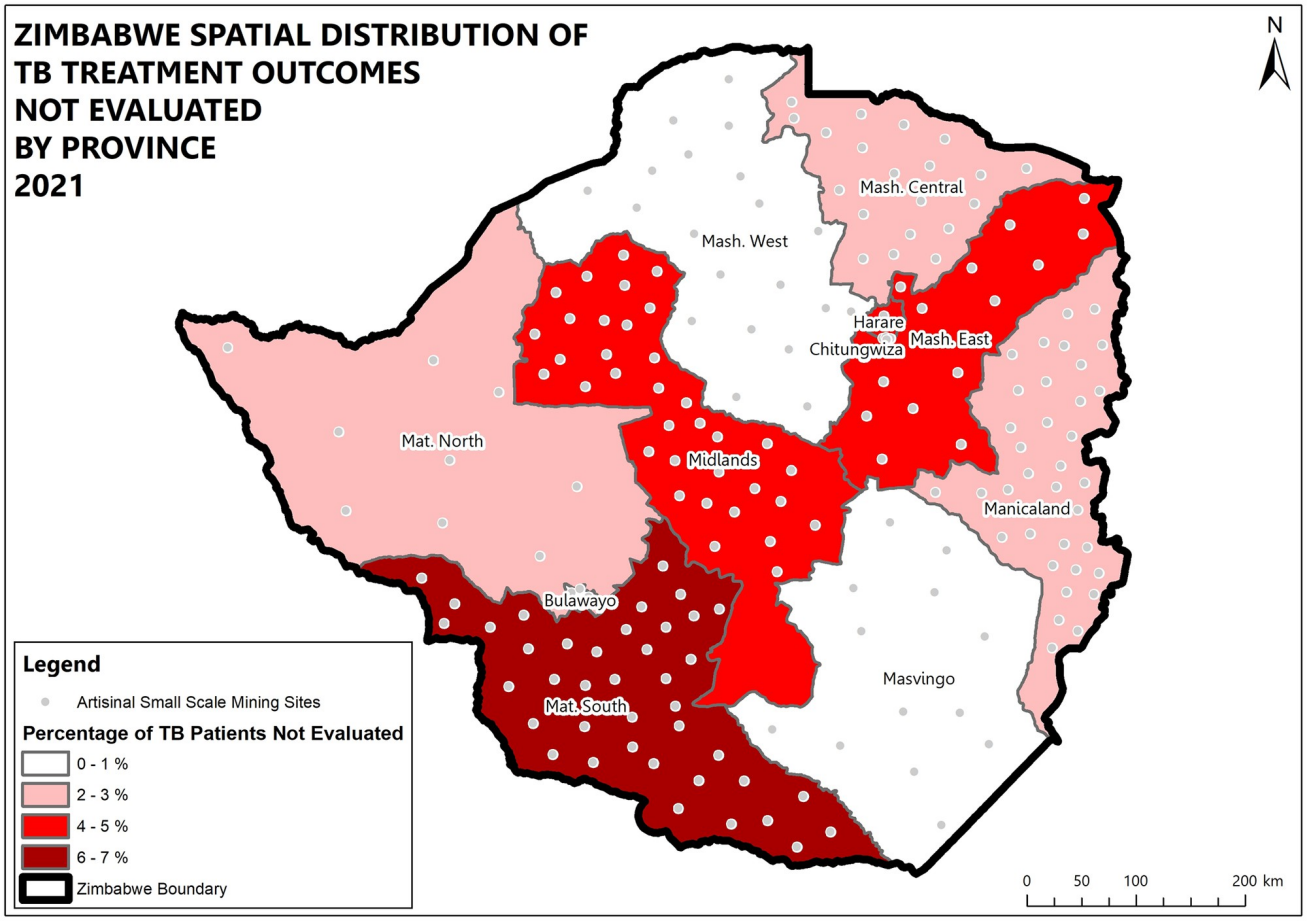
Undesirable treatment outcomes for the 2020 DS-TB cohort in Zimbabwe.

**Fig 5:**
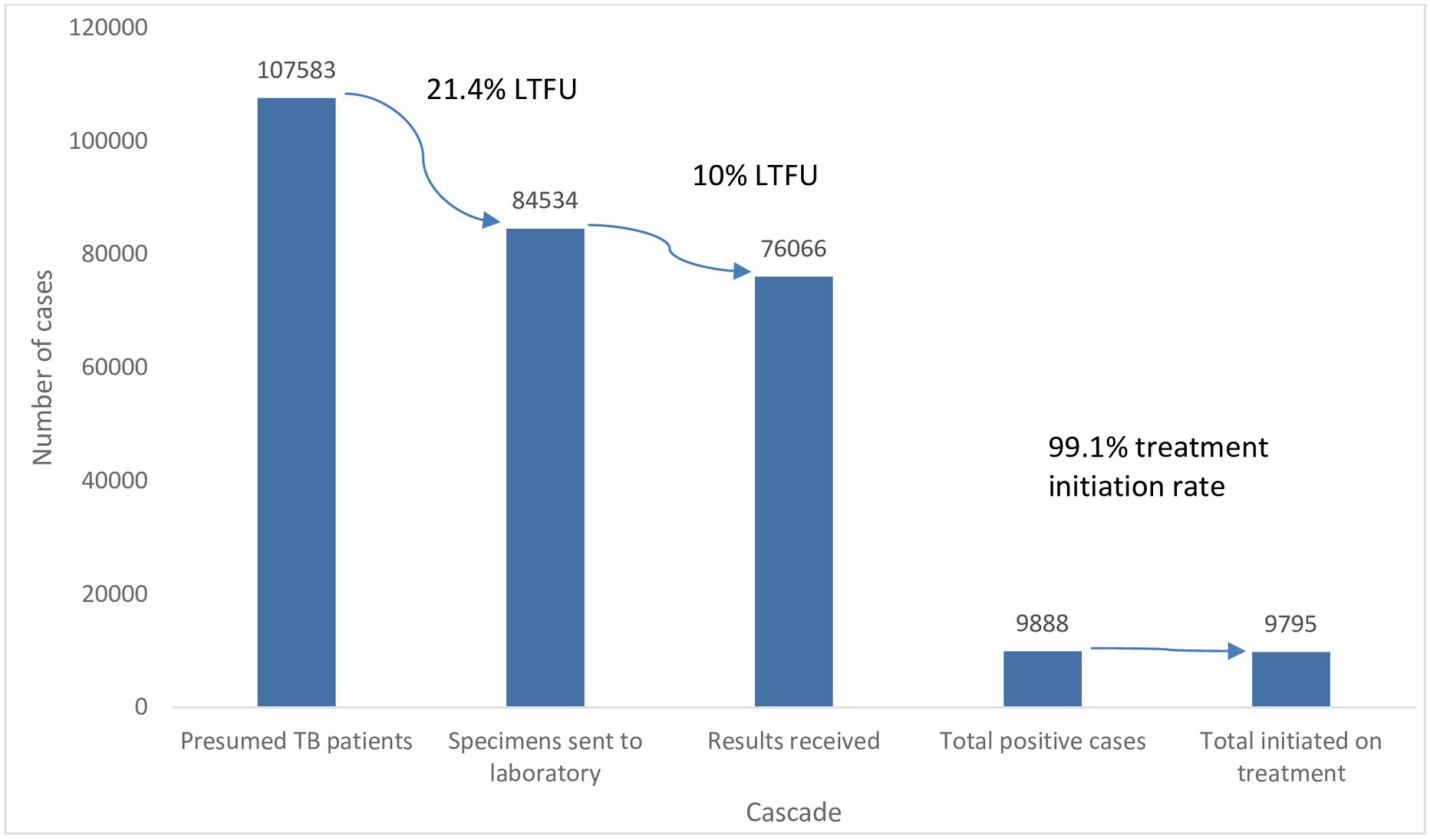
Spatial distribution of DS-TB deaths by province in Zimbabwe 2021.

**Fig 6:**
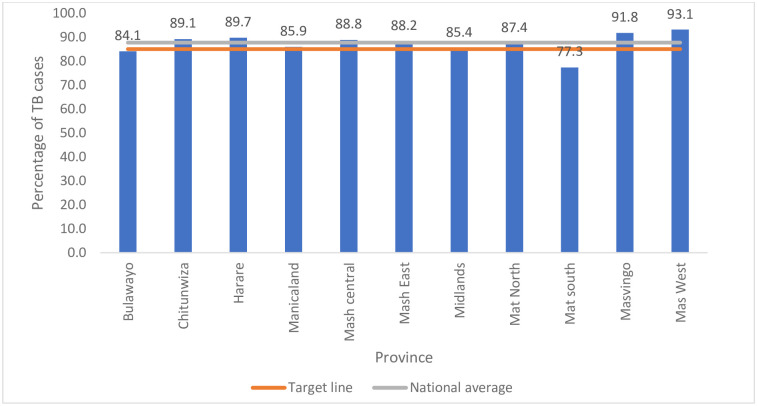
Spatial distribution of DS-TB treatment outcomes not evaluated by province in Zimbabwe 2021.

**Fig 7:**
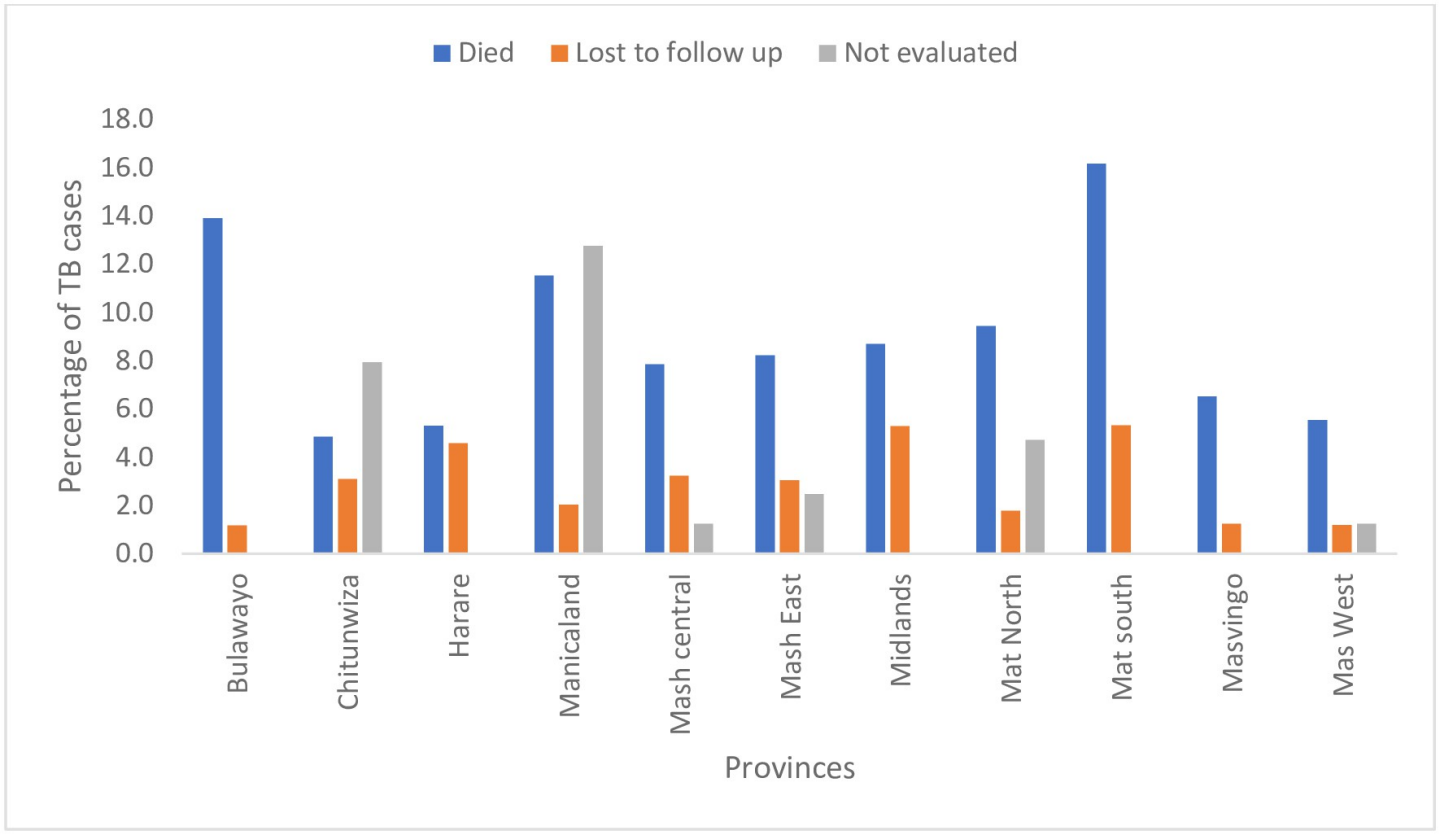
Spatial distribution of DS-TB patients lost to follow-up by province in Zimbabwe 2021.

**Fig 8:**
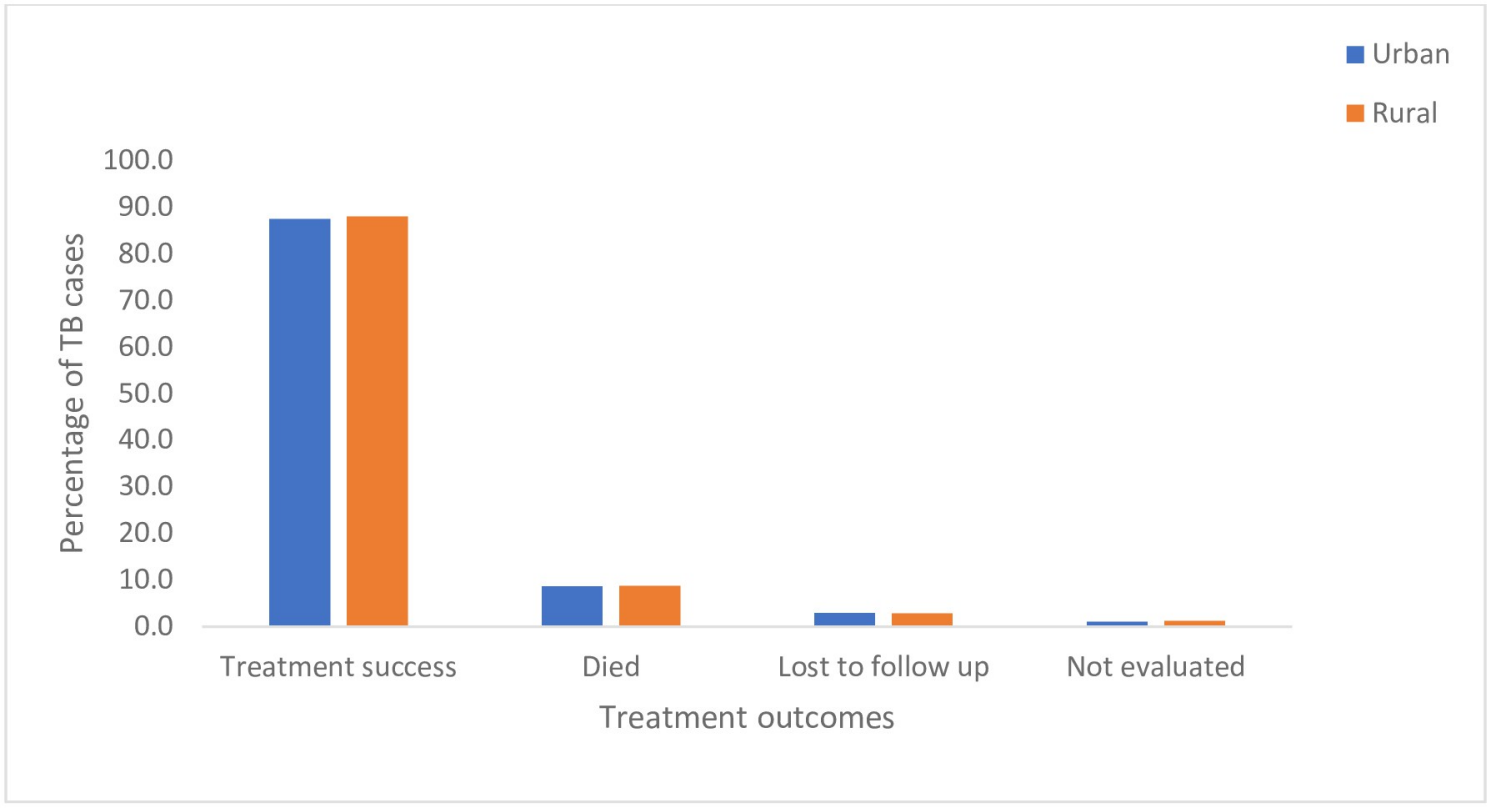
Comparison of TB treatment outcomes between rural and urban provinces in Zimbabwe.

Midlands and Matabeleland South provinces and the highest percentage of patients lost to follow up (5.3%). High death rates were recorded in Matabeleland South (16.2%), Bulawayo (13.9%), and Manicaland (11.5%) provinces while the lowest death rate was recorded in Chitungwiza (4.8%). High death and lost to follow up rates were also prevalent in boarder districts. (Figs 4–8).

Zimbabwe has three urban provinces and eight rural provinces. Treatment outcomes were comparable in both rural and urban provinces. Treatment success rates were 87.4% in urban while rural provinces had a success rate of 87.9%. Similar percentages of people lost to follow up in urban settings (3.0%) and rural provinces (2.9%) were observed (Fig 8).

## Discussion

The key findings of our study were that high death and lost to follow up rates remains a challenge in Zimbabwe and were more prevalent in Matabeleland South, Bulawayo, Masvingo and Manicaland provinces. Treatment outcomes were not evaluated and data quality remains a problem in the national TB control program.

High death rates were recorded in Matabeleland South, and Manicaland provinces. The two provinces are rich in artisanal and small-scale mining activities. The negative outcomes could be attributed to artisanal and small-scale mining (ASM) activities which are mainly unregulated, and there is exposure to dust with minimal to no use of personal protective equipment, and harmful substances like cyanide and mercury. This is further explained by Moyo et al. where ASM communities were found to have high TB/HIV/Silicosis burden, and poor health-seeking behaviours [13] which result in delays in seeking care which contributes to poor treatment outcomes. Since Zimbabwe’s TB epidemic is largely driven by HIV, the unfavourable outcomes could be associated with being coinfected with HIV and not being on antiretroviral therapy [16]. The high death rates and loss to follow-up could be explained by findings from Mambrey et al where they found that ASMs exhibit risky sexual and drug abuse behaviours which are associated with HIV infection and weakened immunity thus resulting in poor outcomes. HIV prevalence in this group was found to be 18% compared to the national prevalence of 12.9% [11, 17]. There is a triple burden of TB, HIV, and silicosis in ASMs, with a TB prevalence of 6.8%, and silicosis 19.0% [11]. ASMs are highly mobile which makes it even hard to follow up and increases transmission within their communities [14]. There are no health facilities within artisanal and small-scale mining communities which also compounds the poor health seeking behaviour. The mining environments are crowded and ASMs are mainly of low socioeconomic status, these factors are associated with poor TB treatment outcomes [12, 18]. Bulawayo province houses major referral centres and centres for excellence for TB services in the southern region. The high death rates in this province could be explained by the fact that they attend to complicated cases from surrounding areas like Matabeleland south province who would have already delayed health seeking.

The proportion of outcomes not evaluated was high. Some reasons given for not evaluated treatment outcomes suggest a deficit in quality data management, analysis and use within the system at facility level. This is supported by the finding from key informants that data can be entered by inexperienced individuals. If data quality management was practiced some of the avoidable errors could be identified before data is sent to the repository. The pre-diagnosis LTFU could have contributed to the estimated 12981 TB cases missed in 2020, and a study by Murongazvombo et al, indicated that being registered at a facility further than 2km from laboratory services or a rural health facility were associated with pre-diagnosis LTFU [19, 20]. Missed TB cases will increase disease burden since an infectious person has the potential to infect 10–15 people in one year [21]. Pre-diagnosis and pre-treatment loss to follow up was also noted in studies by Murongazvombo et. al., and Chadambuka et. al. [20, 22]. Failure to complete treatment may result in TB drug resistance and thus an increase in DR-TB which is costly to manage. The high deaths and loss to follow-up were more prevalent in the two provinces that house the country’s two most active border posts Beitbridge and Nyamapanda. As soon as patients feel better, they then go back to the neighbouring South Africa and Mozambique where they are employed. Some later comeback when they fall ill again and treatment outcomes will then be unfavourable.

Data quality includes completeness and whether data was entered correctly or not. The TB reporting system in Zimbabwe is largely paper based and that could explain the inconsistencies in the data. However, data completeness did not improve in Ireland with the shift from paper-based systems to electronic systems [23]. Another study found that data discrepancies were as a result of missing information in the paper-based systems [24]. Missing information could also contribute to loss to follow up when the missing data includes contact details. Literature also highlighted poor knowledge, negative attitude and data entry errors as reasons for data inconsistencies [24–26]. Data entry errors were reported from key informant interviews. Inadequate data quality can also contribute to underreporting of TB cases globally, affect resource estimates and is therefore important to record and report accurate and good quality data [27].

### Limitations

The outcomes presented may not be a true representation of the true outcomes because a high proportion of outcomes were not evaluated. We could not verify the data reported in DHIS2.3. Challenges with data quality also affect interpretation of the results and to cater for the discrepancies the indicators were calculated with a different denominator from the usual. In this study, number of all forms of TB treatment outcomes analyzed was used as the denominator when calculating proportions of TB outcomes instead of number of all forms of TB cases registered. The difference in calculations also affect measurement of the performance indicators. The use of aggregate data about re-infection on the analysis of treatment outcomes could also interfere with our interpretation of the results and it also limited further analysis to determine factors associated with poor outcomes. Misattribution bias due to use of routine data and the fact that some patients in the “not evaluated” category have been evaluated but not reported.

### Conclusion and recommendations

Data quality and patient loss to follow-up (case holding) remain problems in the TB program. Data quality challenges could be attributed to little to no data analysis and use at facility level. There is need for establishing a cross-border continuum of TB care, Occupational health training, TB screening for ASMs. In addition, strengthening of data related support and supervisions like routine data quality assessments (RDQA) and capacity building for data management to address the reporting challenges and mitigate against high staff turnover/ attrition in most parts of the country. Outdated TB datasets need to be removed or locked to minimize chances of using wrong datasets and to reinstitute quarterly district TB review (data analysis and use) meetings. Active datasets can be clearly labelled to avoid use of wrong versions. It also remains crucial to strengthen patient follow up at all levels within the TB care cascade to minimize loss to follow up and not evaluated outcomes. A study on why LTFU remains high and further analysis to determine factors associated with undesirable outcomes are also recommended.
