# Supplementary material for: Tuberculosis cohort analysis in Zimbabwe: The need to strengthen patient follow-up throughout the tuberculosis care cascade
Source: PLoS One. 2023 Nov 8;18(11):e0293867. doi: 10.1371/journal.pone.0293867 (PMC10631662; doi:10.1371/journal.pone.0293867)
Supplement: Attachment — Submitted filename: TB treatment outcomes in Zim_responses.docx [file pone.0293867.s001.docx]

Dear Editor(s)

Thank you for reviewing our manuscript and for the constructive comments.

Please find the responses to comments raised in our submitted manuscript ‘**Tuberculosis treatment outcomes in Zimbabwe: The need to strengthen patient follow up’**

| **Comment number** | **Editor’s comments** | **Response** |
| --- | --- | --- |
| 1. | Please ensure that your manuscript meets PLOS ONE's style requirements, including those for file naming. | We have followed the style requirements |
| 2. | Please provide additional details regarding participant consent. In the ethics statement in the Methods and online submission information, please ensure that you have specified what type you obtained (for instance, written or verbal, and if verbal, how it was documented and witnessed). If your study included minors, state whether you obtained consent from parents or guardians. If the need for consent was waived by the ethics committee, please include this information. | The study was reviewed and approved by the National TB/HIV Institutional Review Board. All data was deidentified for analysis to ensure confidentiality. We obtained written informed consent from key informants. We obtained permission to conduct the analysis from the Secretary for Health and Child Care, and the Health Studies Office (HSO). |
| 3. | In your Data Availability statement, you have not specified where the minimal data set underlying the results described in your manuscript can be found. PLOS defines a study's minimal data set as the underlying data used to reach the conclusions drawn in the manuscript and any additional data required to replicate the reported study findings in their entirety. All PLOS journals require that the minimal data set be made fully available. | We have attached the minimal dataset  [10.6084/m9.figshare.21737972](https://doi.org/10.6084/m9.figshare.21737972) |
| 4. | Please ensure that you refer to Figure 8 in your text as, if accepted, production will need this reference to link the reader to the figure. | We referenced Figure 8 in the text. |
| 5. | Please upload a new copy of Figure 5 as the detail is not clear. | We have worked on the map and made it clearer. The new version has been uploaded. |
| 6. | We note that Figure 5 in your submission contain map images which may be copyrighted. All PLOS content is published under the Creative Commons Attribution License (CC BY 4.0), which means that the manuscript, images, and Supporting Information files will be freely available online, and any third party is permitted to access, download, copy, distribute, and use these materials in any way, even commercially, with proper attribution. For these reasons, we cannot publish previously copyrighted maps or satellite images created using proprietary data, such as Google software (Google Maps, Street View, and Earth). | We created the maps using ArcGIS software therefore there are no copyright challenges. |
| Abstract  Methods | Statistical analysis is not included. | We calculated means, medians, and frequencies |
| Results | Presumptive TB - on what basis? It would be important to include description in the abstract. | Patients were presumed to have TB based on symptom screening and or chest x-ray |
| Main manuscript  Line 67 | Explain what you mean by unfavourable outcomes due to not being evaluated. | We acknowledge that the statement is vague. The introduction now reads, “Zimbabwe has a high proportion of not evaluated TB treatment outcomes” |
|  | In general, avoid the use of TB cases, and rather refer to People with TB - recommended appropriate phrases according to the words matter by the STOP TB PARTNERSHIP. | The phrase has been edited to People with TB throughout the manuscript. |
| Methods  Lines 85-85 | Provide proportion of cure provided by the public sector. | The public sector provides 95% of all health services. All data analysed in this study were accessed from the public domain. All patients diagnosed with TB from the private sector are referred to the public sector for notification and treatment. |
| Results | Data Quality, need to explain why more cases were evaluated compared to reported. | Some reasons for the discrepancies included 1) facilities evaluated outcomes for patients who were transferred in while the same patients’ outcomes were evaluated at notifying facilities. 2) Not evaluating transfer out cases which in turn were not evaluated by the receiving facilities. 3) Other facilities evaluated outcomes and the findings are either entered in wrong/ outdated datasets or the outcome was not documented. The length of treatment for DR-TB could also contribute to loss to follow up and non-evaluation of outcomes as it may be difficult to follow up patients for long periods. |
|  | TB negative cases are usually not included in TB care cascades. | The study also intended to identify where patients are lost to follow up hence starting the cascade from presumption |
|  | Explain the basis for 99.1% treatment initiation rate. | Of the 9888 people who tested positive for TB, the program was able to initiate 9795 and this equates to a 99.1% treatment initiation rate. The target treatment initiation rate was 100%. |
|  | **Reviewer 1 comments** |  |
| The majority of the discussion section focuses on the geospatial link between provinces with mining communities and death/LTFU in the cohort, and the authors list this finding as a key take-away from their manuscript. The authors should consider the following points: | Currently, the manuscript does not actually present data on mining communities in Zimbabwe. To make their claim compelling, the authors should present data on the distribution of mining communities in Zimbabwe, such as the proportion of each province’s population engaged in ASM or other data that the authors may be aware of. Ideally, the authors would also present statistics supporting the association between ASM and adverse TB outcomes. Otherwise, the hypothesis that ASM is linked to adverse TB outcomes is speculative in this paper—it may be a hypothesis worthy of future study (and, as the authors note, it justified by other prior research), but is not actually evaluated in this manuscript. | -According to the 2012 population census report: artisanal and small-scale miners are largely populated in Midlands (19457), Mashonaland West (13267), Matabeleland South (12153), and Mashonaland Central provinces. The same provinces have a high prevalence of TB in ASMs, high death rates, and loss to follow-up.  -Prevalence of TB in ASMs was 6.8% in Zimbabwe (Moyo, et al, 2022)  -ASMs are highly mobile, work in remote areas with poor access to health services which contributes to late diagnosis which is associated with poor prognosis, and high mobility contributes to high losses to follow-up. |
|  | The claim in the discussion that ASM is linked to adverse TB outcomes because adverse TB outcomes occur more frequently in provinces with high ASM is subject to an ecological fallacy. Because not all individuals living in provinces with high rates of ASM are miners, it is not necessarily correct to claim that ASM causes adverse TB outcomes without doing additional research to support this claim (e.g., directly measuring these outcomes among miners, while controlling for other factors). | We agree that the claim is subject to ecological fallacy. However, the statement was an opinion, or hypothesis and a possible explanation for the findings. A recommendation for further research on the link has been included. |
|  | Typos and unusual syntax occasionally interfered with interpretation of the manuscript, and a careful edit would enhance readability. | We have revised and edited the manuscript |
|  | The authors should harmonize their use of significant digits when reporting proportions and percentages. | We have harmonized our proportions and percentages to one significant figure. |
| Title | The title references “2021”; however, the data reported are from 2019 and 2020. I suggest that the authors either drop the year from the title or change it to better reflect the data presented. | We concur with your guidance and have dropped the year in the topic |
| Abstract  Introduction | Line 27 - The first sentence of the abstract introduction seems to present the primary finding of this paper. Is this information already known (in which case, it would be helpful to describe how this paper builds on this information)? | The introduction has been edited and now reads: ‘A preliminary review of TB treatment outcomes indicated that Mutare City had 22% of outcomes not evaluated in quarter 2 of 2021, and approximately 40% of 2021 outcomes for Harare uniformed forces were not evaluated. The problem persists despite training on data analysis and use. We analysed the TB cohorts to determine the presumptive cascade and the spatial distribution of treatment outcomes in Zimbabwe.’ |
| Methods | Line 31 - It would be helpful to define DHIS2.3 here. | We have defined DHIS 2.3. It means District Health Information Software version 2.3 |
| Results | Line 40-41 - The finding that “only four of 11 provinces were above the national average success rate” simply indicates that these data are skewed: by definition there will always be areas above and below the average; fewer areas above the average only indicates increased skewing of the data. I’m not sure how meaningful this finding is unto itself, unless there is a success rate that is benchmarked to the average that Zimbabwe aims for. | Thank you for the guidance. The statement has been reworded to “seven out of 11 provinces performed below the targeted success rate of 64%. |
|  | Line 43 – see discussion of ASM above | The concerns raised have been addressed in above sections |
| Body Introduction | Line 53 – I think the authors mean to state that “1.5 million people died from TB and 10 million people developed TB in 2020” (per the WHO 2021 World TB Report) (as stated now, it sounds like 15% of the world’s population died from TB in 2020). | We have revised the statement. The statement now reads “1.5 million people died from TB out of the 10 million people who developed TB in 2020” |
|  | Line 55 – the authors should make clear that 85-89% of “TB globally” occurred in the top 30 TB countries, not in Zimbabwe alone. | We have revised the statement. The statement now reads “Zimbabwe remains one of the top 8 countries in Africa on the world’s top 30 list of countries heavily burdened by **TB/HIV and MDR-TB.** The high-burden countries account for 85-89% of people with TB globally” |
|  | Line 57-65 – this paragraph presents methods for TB treatment cohort review. I believe it would fit more appropriately in the methods section | The authors thought it would not fit in the methods section because we did not conduct a cohort review process but rather, we analysed the results of the cohort review process. |
|  | Line 72 – who receives training? | We have qualified the statement so that it is clear. It is healthcare workers involved in TB that receive training. |
|  | Line 76 – I think the authors could further justify why they investigated the spatial distribution of treatment outcomes. Did they have hypotheses about different spatial distributions? Did this analysis serve an administrative or policy purpose, which is why it was pursued? | -The authors investigated the spatial distribution of treatment outcomes to inform programming and aid in crafting targeted interventions to the problem.  -The analysis serves administrative and policy purposes to enable targeted interventions by administrative (geographic) area. |
| Materials and methods | How were “presumptive” TB cases identified? | Patients are presumed based on symptomatic screen and/or chest x-ray |
|  | A variety of different numerators and denominators are used in the analysis, including “deaths of all forms of TB” (line 114) and “new pulmonary bacteriologically confirmed TB cases” (line 117), but it seems as if these disparate groups were added together to attain the overall proportions described in the cascade. Notably, the authors also state that they excluded patients with extrapulmonary TB, but then what is meant by “deaths of all forms of TB”? I am left wondering if these different groups should not have been added together to describe the overall cascade, or if the description of the outcome proportions and exclusions could be further clarified? | -Thank you for the observation the formula was misplaced, and it has been rectified. The correct formula we used included all forms of TB.  -patients with extrapulmonary TB were initially excluded and later included in the final analysis. Leaving the statement as it is, was an oversight on our part. |
|  | Line 80 – the authors discuss characteristics of the administrative province, but the geospatial analysis in the results presents data at the “province district” level. It may be helpful to add a description of the districts as well, or depict only provinces in the maps | Districts have been described in the manuscript. ‘These provinces are further divided into 91 administrative districts from the recent delimitation activities. However, there are still 73 health districts, which offer primary and secondary level care health services.’ |
|  | Line 97-98 – why did the authors choose 2019 to study DR-TB and 2020 to study DS-TB? Were there changes due to COVID-19 that affected reporting of TB cases or function of Zimbabwe’s TB control program, which may affect our interpretation of the DS-TB results (particularly in relation to the pre-COVID DR-TB results)? | Treatment outcomes are evaluated quarterly, and the cohort analyzed is those clients enrolled during the same period the previous year for drug-sensitive TB and the previous two years for drug-resistant TB (because some patients are still on long regimens which may take up to 24 months to complete treatment). The outcomes recorded in the 2021 dataset are outcomes for the 2019 DRTB and 2020 DSTB cohorts. COVID-19 affected the TB program significantly. However, the interpretation of the results may not be affected since the findings for the two cohorts were described/reported separately and were not compared in any way. |
|  | Line 100, 105, 106 – it may be more accurate to state “study outcomes” instead of “study variables” | We have renamed them to study outcomes |
|  | Line 114, 116, 119, 122 – the authors state that they are computing “proportions” (which range from 0-1), but are actually reporting “percentages” (which range from 0-100%). This should be corrected here and in the figures. | The authors have rectified and ensured that all are expressed as percentages. |
|  | Line 139-141 – Why did the authors elect to present maps at the district level, while discussing other results at the provincial level? | We intended to get a more specific picture of whether the undesirable outcomes were contributed by all districts or a few districts within the provinces were not performing well. |
| Results | Consider including an overall demographics table describing the characteristics of included patients | The demographics table has been added and it shows notifications by sex, age, and type of TB |
|  | Line 149 – I am confused as to why there was a discrepancy in the number of patients with outcomes evaluated and cases notified. Reviewing Table 1, it appears that in some provinces, more outcomes were evaluated than cases notified, and in other provinces, the pattern was reversed. My confusion probably arises from my lack of understanding of how “evaluated” and “notification” differ, but I suspect that other readers may be similarly confused. I think it would be helpful to provide additional explanation | Ideally, there should not be discrepancies between notified cases and evaluated outcomes.  An inquiry of possible reasons for the inconsistencies was made through key informant interviews, and some reasons highlighted were --that facilities were not evaluating transfer-out cases which in turn will not be evaluated by the receiving facilities.  - outcomes were evaluated, and the findings were entered in wrong/ outdated datasets  -Outcomes were evaluated but were not documented.  -Some health workers reported to have forgotten to evaluate treatment outcomes due to workload  - |
|  | Line 149 Table 1 – when I add the rows in the “outcomes evaluated” column of Table 1, I get 16115, instead of 16127 (as stated in the text). | Thanks for the correction. We realised that there was a typing and calculation error. Mashonaland west province evaluated 2184 outcomes not 2148 as indicated earlier. The total is now 16151. We rectified the typing and calculation errors. |
|  | Line 155 – it is not correct to say that pre-diagnosis LTFU was 31.4% (achieved by adding 10% to 21.4%), because these two steps have different denominators. I believe the pre-diagnosis LTFU rate that the authors should present is (107583-76066)/ (107583) = 29.3%. | Thank you for the correction. We have rectified it. |
|  | Line 163 – “boarder” should be “border”. It is also not clear to me from the maps that high death/LFTU rates were higher in border districts than in other districts in Zimbabwe. | The spelling has been corrected  -The darker shaded areas are more prevalent in areas that are in and around the border districts. |
|  | Line 165 – why was the DR-TB target 740 cases? | -The target was set based on estimates from the last DR-TB prevalence survey (2016) |
|  | Line 166, 169, 174 – these figure numbers are all off by 1. | We have rectified the figure numbering and now these sections have figure 6,7 and 8 respectively |
|  | Line 175-177 – the authors present results of an inquiry into results, but the methodology for this inquiry is not presented. Were these interviews? Surveys? What questions were asked and what were the hypotheses? Also, it would be helpful to be explicit about what “possible reasons” means. Also, what “other outcomes” (line 176) were evaluated, and why? | -We have included that we conducted key informant interviews to determine why they were discrepancies in the numbers notified and the outcomes evaluated.  -Question asked was “may you please shed light on the discrepancies between number notified and outcomes evaluated?”  - Possible reasons simply meant reasons for discrepancies. We have reworded the statement in the manuscript  -No other outcomes were evaluated. However, the statement meant to indicate that other health facilities evaluated outcomes and entered the findings in wrong datasets or some facilities entered the wrong number notified alongside the outcomes evaluated. |
|  | Line 178-179 – this sentence seems speculative, and probably belongs in the discussion because no data were measured in this study that directly support this statement. | -The authors have moved the statement to discussion section. |
| Discussion | Line 183-184 – there are no data that are actually presented showing high death rates in provinces with ASM (without readers knowing a priori which provinces have high rates of ASM), and it is not clear why the authors focus on ASM in these provinces and not other factors that may be relevant (e.g. HIV prevalence, number/access to health centers, administrative factors affecting reporting of TB data, or others). Therefore, I don’t think the authors should list this as a key finding, unless additional analysis is performed (per above). | -The authors have included data on ASM. Additional discussion points have been included on HIV prevalence, and access to health facilities.  According to the 2012 population census report: artisanal and small-scale miners are largely populated in Midlands (19457), Mashonaland West (13267), Matabeleland South (12153), and Mashonaland Central provinces.  -There is a triple burden of TB, HIV, and silicosis in ASMs. Prevalence of TB in ASMs was 6.8%, HIV and silicosis 18.0% respectively in Zimbabwe (Moyo, et al, 2022)  -ASMs are highly mobile, work in remote areas with poor access to health services which contributes to late diagnosis which is associated with poor prognosis, and high mobility contributes to high losses to follow-up. |
|  | Line 185 (“some reasons given”) – are these the authors’ hypotheses about why non-evaluation rates were high? Or are these the results of inquiries into the reporting system? | -These are results of enquiries into the reporting system (explanations to data discrepancies) |
|  | Line 194-209 – see above regarding concerns about linking ASM with TB outcomes in this study (even if this hypothesis is supported in other studies). | -We have included data on ASM in earlier responses  There are more ASMs in Midlands, Matabeleland South, Mashonaland West, and Mashonaland Central provinces. ASMs are mobile, have poor access to health services. contributes to late diagnosis which is associated with poor prognosis, and high mobility contributes to high losses to follow-up. |
| Limitations  I think it would be helpful for the authors to list and discuss other potential limitations in this study. These include: | -Challenges with data quality (mentioned elsewhere in the manuscript, but not in the limitations section)  -Lack of information about re-infection (which is known to be a major gap in the TB disease care cascade (see for example: <https://pubmed.ncbi.nlm.nih.gov/32072022/>)  -Misattribution bias – could patients in the “not evaluated” category have been evaluated but not reported (e.g. at a different health center)? Thus is there a possibility that use of this outcome measure bias the study’s findings?  - Discordant timing of the study – could inclusion of patients from years before and after COVID-19 onset affect these results or interpretation of the key findings? | We have included challenges with data quality in limitations  -We have included lack of information about re-infection in limitations  -We have included misattribution bias in limitations  -Treatment outcomes are evaluated quarterly, and the cohort analyzed is those clients enrolled during the same period the previous year for drug-sensitive TB and the previous two years for drug-resistant TB (because some patients are still on long regimens which may take up to 24 months to complete treatment). The outcomes recorded in the 2021 dataset are outcomes for the 2019 DRTB and 2020 DSTB cohorts. COVID-19 affected the TB program significantly. However, the interpretation of the results may not be affected since the findings for the two cohorts were described/reported separately and were not compared in any way. |
| Conclusion | The conclusions seem to focus on data quality, which is not extensively discussed in the results or discussion section (there is only 1 sentence in the discussion about data quality). If an appeal for improved data quality is the authors’ key point, then I think it should be further described in the rest of the manuscript, and particularly in the discussion section. | We included discussion on data quality in the manuscript |
| Tables and figures | Figure 3, Figure 4, Figure 5, Figure 7, Figure 8 – I believe these should list “percentage of cases” and not “proportion of cases” | Thank you. We have rectified and changed to percentage of cases |
|  | Figure 4, Figure 8 – the percentages listed atop each of the bars often overlap with adjacent bars and numbers, making them difficult to read. Consider removing these caps or spacing the bars more widely to improve legibility. | We have edited the graphs |
|  | **Reviewer 2 comments** |  |
| Overall | This is a descriptive analysis of routine nationwide tuberculosis data from Zimbabwe. From the manuscript and presented resulted, it can be inferred that the authors analyzed aggregate data rather than case based data. However, this important information is not explicitly mentioned in the methods. Probably the authors assume that all readers are familiar with the format of the district health information system (DHIS) from which their data was obtained. Using aggregate data is an important limitation of this study because characteristics of patients are not available for analysis. If case based data was used, one would have expected detailed analyses of patient characteristics and predictors of unfavorable outcomes. Nevertheless, I believe that health facility characteristics some of which are described in the methods (ownership, level/tier etc) could have enriched the analysis. | -Thank you for the feedback we have included in the methods the following statement, ‘This is a descriptive analysis of routine nationwide aggregate tuberculosis data from Zimbabwe.  -We also noted and included the limitation that aggregate data was used  - We chose to present data by province because the province is the basic management unit in the TB program.  -Analysis by ownership may not apply to our setting because all health facilities are registered under the ministry of health and childcare. Therefore, the government assumes ownership of all health facilities.  -Analysis by tier will be good. However, recommendations will not be targeted since the basic unit of program management are provinces in our setting. We propose an analysis of the provincial data by rural or urban status since management systems in these settings differ. |
|  | Secondly, this analysis included two separate cohorts from two time periods complicating the interpretation of findings. The presented results are also not very clear on the time periods – Table 1 does not mention the time period for the notifications and whether the notifications were drug resistant or sensitive; Figure 2 only mentions the year 2020. Perhaps the authors could focus on drug sensitive TB and conduct a more thorough analysis. | -We included the period for notifications in Table 1 and indicated that it was drug-sensitive TB  -We also included that it is drug-sensitive TB in Figure 2  -We concur with the given suggestion and have focused our analysis on DSTB because we realise the presence of both may introduce difficulties in understanding the message. |
| Additionally, the following issues should be addressed: | The types of TB (extrapulmonary, pulmonary etc) are not presented. Some types of TB are treated for longer periods than others. Depending on when this analysis was conducted, for patients who initiated TB treatment in late 2020 and were to be treated for longer than 6 months may not have completed treatment. These could have been categorized as “not evaluated”. How did the authors ensure that only patients who should have completed treatment at the time of analysis were excluded? | -The formulas highlighted that outcomes for all forms of TB were analysed. In Zimbabwe, TB treatment outcomes for drug-sensitive TB are evaluated 12 months from the time of treatment initiation, for example, those initiated on TB treatment in December 2020 had their outcomes evaluated at the end of 4^th^ quarter of 2021 (i.e., first week of 2022). The analysis was conducted in 2022 and all outcomes analysed were for patients who should have completed treatment. This phenomenon is also described in the introduction section of the manuscript. |
|  | The description of the reporting system for TB data should clearly state that only aggregate data is transmitted based on certain pre-defined indicators. The list of indicators that are routinely reported should be mentioned. | -We have included in the description that ‘only aggregate data is transmitted based on certain pre-defined indicators. Indicators that are routinely reported include: treatment success, cure, treatment completed, failure, death, lost to follow up, and not evaluated rates |
|  | Only a list of TB treatment outcome variables is described yet analyses include presumptive TB cases. The sources and variables for the presumptive analyses are not described. | -We have included the list and source of variables for presumptive analysis in the description of variables. |
|  | There is a mix up in the methods section. The section entitled “study variables” presents a list of how analyses were done. This section should be merged with the “data analysis” section and restructured to be a description rather than a list. | -We have restructured the sections in the manuscript. Thank you for the guidance |
|  | The data analysis section attempts to describe the presumptive TB cascade rather than describing how analyses were conducted. The paragraph talking of this cascade is also confusing with a statement suggesting that smear microscopy should be excluded in the presumptive TB cascade followed by a statement stating that smear microscopy was included. | -Thank you for the observation. We have removed the smear microscopy bit because we realise adding it confuses readers and removing it will not affect our message. |
|  | Additional analyses could have been conducted to explore if some aggregate variables were associated with unfavourable outcomes. In the results and discussions, the authors suggest that residence in certain regions was associated with unfavourable outcomes yet no formal testing of such analyses were performed. | -The study was a descriptive study. In the discussion authors gave their opinion based on literature that indicates the existence of a triple burden of TB/HIV and silicosis in mining communities, and nature of ASMs which are suggestive of poor prognosis in ASMs.  -We included a recommendation for further analysis/ study to determine variables associated with unfavourable outcomes |
|  | The results could have been presented in a sequential manner showing how many participants enter each step of the cascade and how many successfully transitioned to the next step. A flow diagram might have been very useful in illustrating this. The cascade could include steps such as presumptive TB, investigated, diagnosed, treatment initiated, treatment completion. This would assist the authors to better describe their results. | Thank you for the advice  -We have illustrated the steps in a flow chart (Figure 2). |
|  | The first paragraph of the discussion reads like a list rather than a high-level summary of key findings. | -we take note and have restructured it to a more continuous flow of findings. |
|  | This study using routine data had profound limitations that should be discussed elaborately. Currently, the limitations section is too brief. | -We have noted your and other reviewers’ input and have included more limitations. These include:  We have included challenges with data quality, lack of information about re-infection, misattribution bias due to use of routine data. |

|  | **Pulmonary bacteriologically confirmed** | | **Pulmonary clinically diagnosed** | | **Extrapulmonary TB** | |  | |
| --- | --- | --- | --- | --- | --- | --- | --- | --- |
| **Age** | **Male** | **Female** | **Male** | **Female** | **Male** | **Female** | | **Total** |
| <15 | 132 | 126 | 290 | 272 | 47 | 34 | | 901 |
| 15-64 | 5291 | 2570 | 3207 | 1923 | 602 | 427 | | 14020 |
| 65 + | 292 | 145 | 374 | 213 | 77 | 54 | | 1155 |
| **Total** | **5715** | **2841** | **3871** | **2408** | **726** | **515** | | **16076** |

| **Age groups** | **Male** | **Female** | **Total** |
| --- | --- | --- | --- |
| <15 | 2 | 4 | 6 |
| 15-64 | 218 | 120 | 338 |
| 65+ | 7 | 5 | 12 |
| **Total** | **227** | **129** | **356** |
|  |  |  |  |
