# Supplementary material for: Tuberculosis cohort analysis in Zimbabwe: The need to strengthen patient follow-up throughout the tuberculosis care cascade
Source: PLoS One. 2023 Nov 8;18(11):e0293867. doi: 10.1371/journal.pone.0293867 (PMC10631662; doi:10.1371/journal.pone.0293867)
Supplement: Attachment — Submitted filename: TB treatment outcomes_ Response to Reviewers.docx [file pone.0293867.s002.docx]

Responses to comments raised in our submitted manuscript ‘**Tuberculosis treatment outcomes in Zimbabwe: The need to strengthen patient follow up’**

Thank you for reviewing our manuscript and for the constructive comments. Please find below our responses to the comments.

| **Comment number** | **Editor’s comments** | **Response** |
| --- | --- | --- |
| General | Some of the syntax and grammar in the revised version remains difficult to understand, and there are still a few typos in the revised manuscript. I would recommend another read-through to ensure legibility. | Thank you for the feedback. The manuscript has been read through and revised |
| **Introduction**  Line 82 | Would define the acronym DHIS2 (even though defined in the abstract) (also, is this missing a “.3”?) | The acronym has been defined. Thank you for identifying the typo the .3 was missing and has been included |
| Line 96 | Possible to further define “the problem” (several problems have been introduced in this paragraph- would be helpful to specify which problem the authors are referring to) | The problem has been further defined and the sentence now reads ‘This prompted an assessment of the TB treatment cascades and spatial distribution of treatment outcomes to inform programming and aid in crafting targeted interventions to the problems of unevaluated TB treatment outcomes).’ |
| **Methods**  Line 101 -103 | The data on Zimbabwe’s TB burden is already presented in the introduction; probably not necessary to repeat here | Thank you for the guidance. The information/data has been removed |
| Line 113 | Would clarify what the numbers after the provinces refer to. I think these are populations? | the numbers after the populations are populations. The sentence has been revised to read ‘Artisanal and small-scale miners are largely populated in Midlands province with a population of 19457 ASMS, followed by Mashonaland west 13267, Matabeleland South 12153, and Mashonaland central provinces’ |
| Line 122 | Would define DHIS2 at its first use (in line 82) | DHIS2 has been defined at line 82. |
| Line 149 and throughout equation | would be consistent in how denominators are listed (“No. of all forms of TB cases registered” vs “Number of TB cases registered” vs “No. of TB cases registered”) | The formulas have been revised. We also note that we had written the standard denominator for the indicators versus the actual denominators we used in our calculation. |
| Line 162 | It would be helpful to discuss who the key informants were, how they were contacted, and how these data were analysed. | Thank you for the guidance. The paragraph has been revised and it now includes who the key informants were (TLCs, monitoring and evaluation officers, health information officers, TB focal persons), they were contacted through phone calls and face-to-face discussions. A thematic analysis of the responses was conducted. |
| **Results**  Line 188 | I’m still confused as to how there were more outcomes evaluated than cases notified. If the authors’ point is that this discrepancy is an indication of lack of data quality, then I think this point should be explicitly stated. | It has been explicitly stated that lack of data quality was the main explanation for the data discrepancy per your guidance. Other reasons why more outcomes were evaluated have also been stated under responses from key informants |
| Figure 5 - 8 | The figure captions list that these outcomes are represented by province and district. however, it looks like only districts are shown in the map. It would be helpful to show outcomes by provinces, because the provincial level is extensively discussed in the text. | New provincial maps have been constructed and attached |
| Figure 5 -8 | Recommend changing proportion to percentage | Maps now indicate that they are percentages, not proportions as earlier indicated |
|  | The authors mention key informant interviews in the methods, but results of these interviews aren’t explicitly reported in the results. In my mind, these results are potentially some of the most important for the authors to bring up, because they seem like they have potential to provide important details about data quality problems. | Thank you for the guidance. The qualitative results of the key informant interviews have been included in the text. |
| **Discussion** | I’m still skeptical of the claim that “The key findings of our study were that high death and lost to follow-up rates were prevalent in provinces with artisanal and small-scale mining (ASM) activities”. Currently, the results section does not mention ASM activities, their distribution within provinces, or any statistical tests that show that provinces with high ASM activities were more likely to experience higher death and loss to follow-up than other provinces. I acknowledge the author’s response that the statement was an opinion, or hypothesis and a possible explanation for the findings”. However, in that case, and without any explicitly detailed evidence supporting this claim, I don’t think the authors should state that it is their “key finding”, as currently remains in the revised text. | The provincial maps that depict a link between provinces with ASM activities and the unfavourable treatment outcomes. The statement was removed from key finding. |
|  | Because much of the discussion with the link between provinces with high ASM and adverse TB outcomes, it would be helpful to include a map of the provinces of Zimbabwe, ideally coloured by ASM concentration to illustrate the overlap with TB outcomes (which also ideally would be represented at the provincial level). | The initially submitted district maps have been replaced with the newly constructed provincial maps. |
|  | It would also be helpful to discuss the results of the key informant interviews | A discussion on the results of the key informant interviews has been incorporated into the discussion |
| **Limitations**  Line 314 | It would be helpful to define what “the usual calculations” are and how the authors’ calculations are different. | The section has been revised and now reads ‘Challenges with data quality also affect the interpretation of the results and to cater for the discrepancies the indicators were calculated with a different denominator from the usual. In this study, the number of all forms of TB treatment outcomes analyzed was used as the denominator when calculating the proportions of TB outcomes instead of the number of all forms of TB cases registered.’ |
|  | **Reviewer #2** |  |
|  | The authors have satisfactorily addressed reviewer comments. I still find the title confusing – Loss to follow up in the title sounds is tied to treatment outcomes which is not the case in the manuscript which has identified per-diagnosis loss to follow-up as one of the notable results. Additionally, some of the figures – the maps are not very clear. | -Thank you for the feedback. The title has been revised to ‘**Tuberculosis cohort analysis in Zimbabwe: The need to strengthen patient follow up throughout the TB care cascade’**  **-**The maps have been revised and have constructed provincial maps instead of district maps |
